# Current advances in molecular subtyping using multilocus variable number of tandem repeat analysis of *Salmonella* Enteritidis and *Salmonella* Typhimurium in Egyptian chickens

**DOI:** 10.14202/vetworld.2020.2252-2259

**Published:** 2020-10-28

**Authors:** Wafaa M. M. Hassan, Ashraf A. Abd El Tawab, Sara M. El-Shannat

**Affiliations:** 1 Reference Laboratory for Quality Control on Poultry Production, Animal Health Research Institute, Dokki, Giza, Egypt; 2 Department of Bacteriology, Immunology, and Mycology, Faculty of Veterinary Medicine, Benha University, Benha, Egypt; 3 Department of Microbiology, Animal Health Research Institute, Marsa Matruh, Egypt

**Keywords:** lipopolysaccharides, multilocus variable number of tandem repeat analysis, *Salmonella*, serotyping

## Abstract

**Aim:**

This study aimed to characterize the genetic diversity, evolutionary level, and prevalence of genotypes of common isolates of *Salmonella* (*Salmonella* Enteritidis and *Salmonella* Typhimurium). Using one of the most advanced molecular recognition techniques, multilocus variable number of tandem repeat analysis (MLVA), we characterized the genotype and prevalence of *S*. Enteritidis and *S*. Typhimurium.

**Materials and Methods:**

One hundred and twenty-five internal organ samples were collected from the major chicken slaughterhouses in Egypt, and *Salmonella* species were isolated. PCR was utilized to amplify the *IE-1* and *Flic-C* genes to identify *S*. Enteritidis and *S*. Typhimurium DNA, respectively, from *Salmonella* isolates. MLVA was applied on nine samples of *S*. Enteritidis DNA and three samples of *S*. Typhimurium DNA. Six variable number tandem repeat (VNTR) loci (Sal02, Sal04, Sal06, Sal10, Sal20, and Sal23) were amplified.

**Results:**

Of the examined samples (n=125), a total of 12 isolates (9.6%) were either identified as Enteritidis or Typhimurium. PCR-mediated amplification of *IE-1* and *Flic-C* revealed that 75% (n=9) of the 12 *Salmonella* isolates were *S*. Enteritidis and 25% (n=3) were *S*. Typhimurium. The six loci amplified through MLVA had allelic diversity. The most discriminatory heterogenic locus for *S*. Enteritidis was Sal20. Sal04 and Sal23 were the most discriminatory heterogenic loci for *S*. Typhimurium. VNTR allelic profile analysis revealed nine unique genotypes for *S*. Enteritidis and three for *S*. Typhimurium.

**Conclusion:**

This study was the first to use MLVA analysis to identify *S*. Enteritidis and *S*. Typhimurium strains isolated from chickens in Egypt. The molecular typing data reported herein allowed us to characterize the genotypes of *S*. Enteritidis and *S*. Typhimurium that are most prevalent in Egyptian chickens. Moreover, this epidemiological information provides valuable insight on how to prevent disease transmission. Moreover, our methods provide an alternative to traditional serotyping techniques that may produce inaccurate strain identifications for organisms with rough lipopolysaccharide structures.

## Introduction

*Salmonella enterica* serotypes Enteritidis and Typhimurium are two of the most famous zoonotic strains of *Salmonella* worldwide [1]. The serotype *Salmonella* Enteritidis possesses one of the most prominent risks for severe economic danger in Europe and many countries throughout the world [2]. The serotype Typhimurium is considered a major cause of human and animal salmonellosis [3]. A multi-country outbreak of *S*. Enteritidis confirmed through whole-genome sequencing (WGS) analysis is currently ongoing. There are currently 314 confirmed cases in Austria, France, Ireland, Luxembourg, and the United Kingdom. Besides, Austria, Belgium, Denmark, the Netherlands, Norway, and the United Kingdom reported collectively 21 probable and 50 historical probable cases. Confirmed cases belong to three closely related genetic clusters. Possible cases have been related to the multilocus variable number tandem repeat (VNTR) multilocus variable number of tandem repeat analysis (MLVA) profiles 2-11-3-3-2 and 2-12-3-3-2. However, 1 additional confirmed isolate has an MLVA profile of 2-9-3-3-2 [4].

Poultry and poultry byproducts are serious vehicles in infection transmission to consumers [5]. Moreover, chickens infected with *S*. Enteritidis play a role in infection spread through trans-ovarian transmission during the stage of egg development [6]. Most reported cases of *S*. Typhimurium infection are also related to infected food products; few cases are reported as a result of direct contact with infected animals or contaminated water or environment [7,8].

Due to a lack of distinctive genotype properties between *S*. Enteritidis and *S*. Typhimurium isolate,it is possible that they have evolved from the same ancestor clone. Determining an accurate clone is complicated by the fact that the *Salmonella* species is not asexual and the final end product of recombination replacement can result in a large number of highly diverse genotypes under the same clonal complex. The clonal diversity of bacteria varies according to the extent of recombination that can vary depending on bacterial species [9]. As MLVA and WGS are not utilized in all EU/EEA countries, other European Union countries may be inadvertently affected by this outbreak. This underscores the need for the use of MLVA analysis to identify comorbidities related to the ongoing outbreak. Performing WGS analysis on isolates associated with the outbreak on MLVA profiles would further confirm their participation in the ongoing outbreak [4].

In Egypt, there is a lack of genetic-relatedness data collected from *S*. Enteritidis and *S*. Typhimurium isolated from chickens. It is imperative to use highly advanced molecular subtyping techniques such as MLVA, to accurately identify strains with rough lipopolysaccharide structures. 

A combination of MLVA with other molecular techniques, such as pulsed-field gel electrophoresis (PFGE), ribotyping, and phage typing, provide clear data that can accurately identify the outbreak strain and distinguish it from epidemiologically unrelated isolates [10-12].

The three methods previously noted have been proven incompetent in *Salmonella* typing when not used in conjunction with techniques such as MLVA. Many reports demonstrate that PFGE is considered insufficient as a tool for molecular typing of *Salmonella* species, mainly due to poor reproducibility in different aboratories. Ribotyping also has low discrimination power and is not useful in the current epidemiological investigation and surveillance study of the ongoing outbreak [13-16]. The use of phage typing is not ideal because it revealed that some *S*. Enteritidis strains could not be typed due to phage conversion among strains [17].

For these reasons, MLVA is considered the current method of choice for genotyping *S. enterica* (*S*. Enteritidis and *S*. Typhimurium) in most laboratories. MLVA is fast, highly reproducible, cost-effective, can be used with small amounts of DNA, can discriminate between clinical isolates, and allows for systematic analysis as it opens a way for data standardization through processing and development of web-based resources for database query [18,19]. MLVA analyzes the variation in the numbers of tandem repeated sequences in DNA at multiple genomic loci [20]. This means that each target region is individually amplified using specific primers that anneal to the flanks of the repeat-containing region. Determination of the amplicon sizes by electrophoresis on agarose gels enables the number of tandem repeat units present at each locus to be deduced [21-23].

The application of the MLVA technique using Interspersed Repetitive Units-Variable Number of Tandem Repeats for the identification of *S*. Enteritidis and *S*. Typhimurium strains isolated from chickens provides accurate data to appropriately identify outbreak strains and distinguish them from epidemiologically unrelated isolates. Furthermore, the MLVA technique can help in determining the prevalence of different *Salmonella* genotypes.

This study aimed to characterize the genetic diversity, evolutionary level, and genotype prevalence of the most predominant isolates of *Salmonella* (*S*. Enteritidis and *S*. Typhimurium) in Egyptian chickens. Using the advanced MLVA technique allowed us to decipher the genotypes and spread of *S*. Enteritidis and *S*. Typhimurium in Egypt.

## Materials and Methods

### Ethical approval

No ethical approval was needed to perform this study. However, the samples were treated according to the national and international criteria. 

### Sampling

One hundred and twenty-five random samples from broiler chickens tissues (internal organs) were collected from different slaughterhouses in Egypt for examination, in the period from December 2017 to October 2019.

The samples were collected following the protocol recommended by the International Commission on Microbiological Specification for Food [24]. The samples were transported on ice to the reference laboratory for quality control on poultry production (Animal Health Research Institute, Dokki, Giza, Egypt). The samples were sealed in sterile bags and stored at −86°C.

### Bacteriological examination

The samples were treated according to the method described by standard ISO 6579:2002 [25] and CDC manual [26].

### Isolation of *Salmonella* species from chicken tissue samples

In brief, 5 g of tissue samples, which included intestine, liver, kidney, heart, and spleen, were thawed at room temperature under aseptic conditions. The samples were then macerated into fine pieces using sterile blades, homogenized using a sterile pestle and mortar, added to 45 mL of buffered peptone water (HiMedia, India), and then incubated at 37°C for 18 h. After the incubation, either 0.1 mL or 1 mL of pre-enriched aliquots were transferred into 10 mL Rappaport and vassiliadis broth for the enrichment and incubated at 42°C for 24 h (HiMedia, India). Finally, the enriched aliquot samples were seeded on MacConkey and xylose-lysine-deoxycholate (XLD) agar [27] and incubated at 37°C for 24-48 h for the observation of the growth of typical *Salmonella* colonies (HiMedia, India), (i.e., pink colonies with a black center on XLD plates and colorless colonies on MacConkey plates).

### Extraction of *Salmonella* genomic DNA

Genomic DNA was obtained from *Salmonella* subcultures from XLD (HiMedia, India), plates according to manufacturer’s instructions from the QIAamp Blood and Tissue Kit for Gram-Negative Bacteria (Qiagen®, Hilden, Germany). Briefly, colonies (2-3) were harvested in a microcentrifuge tube by centrifuging for 10 min at 5000× *g* (7500 rpm). Supernatants were subsequently discarded and the remaining pellet was resuspended in 180 μL buffer ATL. 20 μL of proteinase K was added and the mixture was incubated for 2 h at 56°C to allow for extensive bacterial lysis. DNA was eluted from the QIAamp mini spin columns with 100 μL of buffer to increase the concentration of the recovered DNA. The concentration of DNA was measured using UV absorption with a spectrophotometer (BMG Labtech, Germany) at a wavelength of 260 nm. The DNA was stored at −20°C for further analysis.

### Molecular identification of *S. Enteritidis* and S. Typhimurium DNA based on *IE-1* and *Flic-C* genes

PCR was used to amplify the *IE-1* and *Flic-C* genes, which are specific for *S*. Enteritidis and *S*. Typhimurium, respectively. A 316- and 432-bp fragment containing *IE-1* and *Flic-C* gene sequences was amplified by PCR. Primers specific for *IE-1* were: *IE-1*/F (5'- AGT GCC ATA CTT TTA ATG AC -3') and *IE-1*/R (5' - ACT ATG TCG ATA CGG TGG G -3'). Primers for *Flic-C* were: *Flic-C*/F (5'- CCC GCT TAC AGG TGG ACT AC -3) and *Flic-C*/R ('5'- AGC GGG TTT TCG GTG GTT GT-3). These primers have been found to be specific and more reliable for the differentiation of *S*. Typhimurium from other members of the *Salmonella* species [28]. DNA samples were subjected to two differential amplification reactions in two separate tubes. The *S*. Enteritidis specific amplification reaction mixtures were identical to those of *S*. Typhimurium with the exception of the primers targeting *IE-1* instead of *Flic-C*. PCR reactions were carried out in 25 μL containing 5 μL of 5× PCR master mix (Jena bioscience®, Germany), 3 μL of each primer (forward and reverse) at a working concentration of 10 pmol/μL, (Metabion International AG®, Germany) 2 μL of DNA template, and 12 μL ddH2O. For *MTBC* identification, the primer set *IE-1*/F, *IE-1/*R, and *Flic-C/*F*, Flic-C/*R was used according to the following parameters: Initial denaturation at 95°C for 5 min, followed by 30 cycles at 95°C for 1 min, annealing at 58°C for 1 min, extension at 72°C for 30 s, and a final extension at 72°C for 7 min [29]. The amplification products were resolved by electrophoresis on a 1.5% agarose gel, followed by ethidium bromide staining (0.5 μg/mL) and examination under UV light.

### MLVA

Literature was extensively reviewed to identify primer sequences for *S. enterica* loci with potential to distinguish *S*. Enteritidis and *S*. Typhimurium strains. Six polymorphic tandem repeat loci, which vary in size from 107 to 250 bp, were expected to have potential to distinguish the two serovars [30]. In this study for MLVA analysis, six VNTR (0681, 0764, 0789, 2053, 4301, and 4774) primer pairs were used (Table-1) [31]. The PCR reaction was performed in a total volume of 25 μL per reaction with the following reagent concentrations: 5 μL of 5× PCR master mix (Jena bioscience®, Germany), 3μL of each primer (forward and reverse) at a working concentration of 10 pmol/μL, (Metabion International AG®, Germany) 12 μL ddH2O, and 2 μL of genomic DNA as a template. PCR was conducted (Biometra, Germany) with the following cycling parameters: Initial denaturation at 96ºC for 5 min, followed by 30 cycles at 96°C for 30 s, annealing at 62°C for 1 min, extension at 72°C for 1 min, and final extension at 72°C for 10 min. Positive and negative controls were included in each set of reactions and consisted of the same reaction mixtures with genomic DNA of *S*. Enteritidis and *S*. Typhimurium reference strains as the template for the positive control and no template DNA in the negative control. The PCR fragments were analyzed by gel electrophoresis using 2% agarose stained with ethidium bromide for each locus. Sizes of amplicons were estimated by comparison with 50 bp markers (Jena bioscience®, Germany), and the number of repetitive units was determined. The genomic sequences of *S*. Enteritidis and *S*. Typhimurium (ATCC 13076 and ATCC 14028, respectively) were also used as reference strains and were evaluated to obtain the amplicon sequence for each locus [32].

**Table 1:** Primer sequences and the size of the repeat units of the VNTR loci in this study.

VNTR locus	VNTR locus alias	VNTR locus size (bp)	PCR Primer sequences (5’-3’)
0681	Sal 02	6	(F) GGA AAG ACT GGC GAA CAA AT
			(R) TCG CCA ATA CCA TGA GTA CG
0764	Sal 04	20	(F) TCG CAC AGA TGA CCA ATT TT
			(R) GAT CGA CGC TCA CTG CTT C
0789	Sal 06	6	(F) TTGGTCGCGGAACTATAACTG
			(R) CTTCGTCTGATTGCCACTCC
2053	Sal 10	12	(F) AAGCGACGTTCTTCTGCAAC
			(R) TGGAATATGATGGCATGACG
4301	Sal 20	3	(F) CAGCCGACACAACTTAACGA
			(R) ACTGTACCGTGCGCGTTT
4774	Sal 23	12	(F) CCCGCACACTAAGGAGAGAC
			(R) ACCGCGTTAGTGGCTAACAT

List of primers used for *S.* Enteritidis and *S.* Typhimurium strains typing obtained from [31]. A total of six loci were selected for amplification of DNA from *Salmonella* isolates

### Determination of tandem repeats

The number of tandem repeats in each amplified MLVA locus was estimated based on the amplified MLVA loci size and the size of the flanking region using the formula in Table-2.

**Table 2:** Description of each VNTR locus, repeat unit size, flank size, tandem repeat copy number, number of alleles, and fragments length size.

Locus	Repeat unit size (bp)	No. of alleles		Tandem repeat coy number		Fragment length (bp)		Flank size (bp)	Description of each
			
		SE	Tm	SE	Tm	SE	Tm		VNTR locus#
Sal 02	6	4	2	1, 2, 3, 4	4, 5	95, 101, 107, 113	113, 119	89	(6×1)+89, (6×2)+89…etc.
Sal 04	20	3	3	1, 2, 3	1, 2, 3	174, 194, 214	174, 194, 214	154	(20×1)+154, (20×2)+154….etc.
Sal 06	6	2	2	2, 3	5, 6	156, 162	174, 180	144	(6×2)+144, (6×3)+144
Sal 10	12	3	2	1, 2, 3	3, 4	184, 196, 208	208, 220	172	(12×1)+172, (12×2)+174….etc.
Sal 20	3	6	2	8, 10, 11, 12, 13, 14	16, 17	169, 175, 178, 181, 184, 187	193, 196	145	(3×8)+145, (3×10)+145…etc.
Sal 23	12	3	3	3, 4, 5	4, 5, 6	250, 262, 274	264, 274, 285	214	(12×3)+214, (12×4)+214….etc.

# Each VNTR locus was described as (repeat unit size×copy number) + size of the partial repeat. SE=*Salmonella* Enteritidis, Tm=*Salmonella* Typhimurium

PCR assays for the six loci were repeated and compared within and between gels to ensure consistent estimation of size and tandem repeat copy number.

## Results

**Figure-1:**
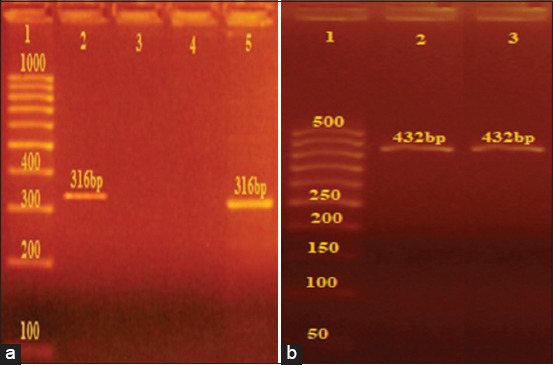
PCR- identification of *Salmonella* Enteritidis and *Salmonella* Typhimurium DNA based *IE-1* sequences and allele-specific *Flic-C* gene. (a) DNA extraction from *Salmonella* isolate. Amplicons obtained with *IE-1* (lanes 2, 5) with the size of 316 bp. Lanel: 100 base pair ladder, (Jena bio science®). (b) DNA extraction from *Salmonella* isolate. Amplicons obtained with *Flic-C* (lanes 2, 3) with the size of 432 bp. Lanel: 50 base pair ladder, (Jena bio science®).

PCR-mediated detection of *S*. Enteritidis and *S*. Typhimurium DNA from isolates based on the presence of *IE-1* and *Flic-C* genes 12 *Salmonella* isolates (9.6% of the samples collected) were identified in this study. All DNA extracted from bacterial isolates was subjected to PCR amplification of target sequences specific for *S*. Enteritidis (*IE-1*) and *S*. Typhimurium (*Flic-C*) (Figure-1). PCR based *IE-1* gene and *Flic-C* identification for *Salmonella* isolates revealed that 9/12 of the *Salmonella* isolates were *S*. Enteritidis and 3/12 were *S*. Typhimurium (Table-3).

**Table 3:** Results of PCR-based *IE-1* sequences and allele-specific *Flic-C* gene method versus culture on MacConkey and XLD agar.

Isolate No.	Culture media		DNA from *Salmonella* isolates	
	
	MacConkey agar	XLD agar	*IE-1*	*Flic-C*
1	+ve	+ve	+ve	–ve
2	+ve	+ve	+ve	–ve
3	+ve	+ve	+ve	–ve
4	+ve	+ve	+ve	–ve
5	+ve	+ve	+ve	–ve
6	+ve	+ve	+ve	–ve
7	+ve	+ve	+ve	–ve
8	+ve	+ve	+ve	–ve
9	+ve	+ve	+ve	–ve
10	+ve	+ve	–ve	+ve
11	+ve	+ve	–ve	+ve
12	+ve	+ve	–ve	+ve

### Genotyping of S. Enteritidis and S. Typhimurium using MLVA

MLVA was applied to 12 samples of *Salmonella* DNA which was classified into two groups. Based on the amplification of the six VNTR loci, the first group consisted of nine samples which were classified as *S*. Enteritidis and the second group consisted of three samples that were classified as *S*. Typhimurium. These six polymorphic loci identified 12 unique MLVA genotypes (Table-4). Nine unique genotypes were specific to *S*. Enteritidis ***(***A, B, C, D, E, F, G, H, and I) while three unique genotypes (J, K, and L) were specific to *S*. Typhimurium.

**Table 4:** The VNTR allelic profiles of the *S.* Enteritidis and *S.* Typhimurium in Egypt.

Locus	VNTR copy number for six polymorphic loci (12 unique multilocus variable number of tandem repeat analysis type A-L)												Tandem repeat copy number/locus	
	
	A	B	C	D	E	F	G	H	I	J	K	L	*S.* Enteritidis	*S.* Typhimurium
0681	1	2	2	3	3	4	4	4	4	5	4	5	1, 2, 3, 4	4, 5
0764	1	1	1	1	1	1	2	3	3	3	2	1	1, 2, 3	1, 2, 3
0789	2	3	3	2	2	2	2	3	3	5	5	6	2, 3	5, 6
2053	1	1	1	2	1	3	3	1	2	4	4	3	1, 2, 3	3, 4
4301	12	14	13	12	12	11	10	8	12	17	16	16	8, 10, 11, 12, 13, 14	16, 17
4774	3	3	3	4	4	3	4	5	5	6	5	4	3, 4, 5	4, 5, 6

*S.* Enteritidis=*Salmonella* Enteritidis, *S.* Typhimurium=*Salmonella* Typhimurium, VNTR=Variable number tandem repeat

**Figure-2:**
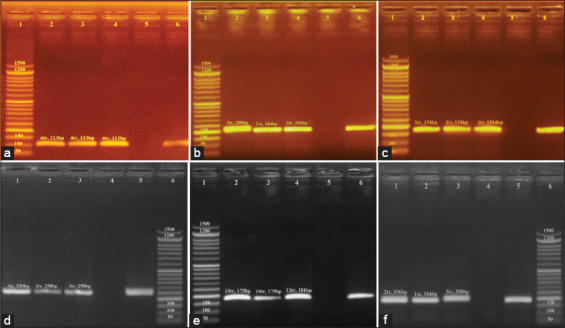
The results of multilocus variable number of tandem repeat analysis typing, based on DNA extracted from grown *Salmonella* Enteritidis isolates. (a) MLVA typing of DNA from grown *S*. *enteritides* isolates using the 0681 locus (Lane 2, 3, and 4), with a tandem repeat size of 6 base pairs. Lanel: 50 base pair ladder; Lane 2, 3 and 4: Amplicon obtained based on 0681 locus with fragment length of 113 bp (4tr); Lane 5, and 6: negative and positive control respectively. (b) MLVA typing of DNA from grown *S. enteritides* isolates using the 2053 locus (Lane 2, 3, and 4), with a tandem repeat size of 12 base pairs. Lane1: 50 base pair ladder; Lane 2, 3 and 4: Amplicon obtained based on 2053 locus with fragment length of 208 bp respectively; Lane 5, and 6: negative and positive control respectively. (c) MLVA typing of DNA from grown *S. enteritides* isolates using the 0764 locus (Lane 2, 3, and 4), with a tandem repeat size of 20 base pairs. Lanel: 50 base pair ladder; Lane 2, 3 and 4: Amplicon obtained based on 0764 locus with fragment length of 174 bp (1tr); Lane 5, and 6: negative and positive control respectively. (d) MLVA typing of DNA from grown *S. enteritides* isolates using the 4774 locus (Lane 2, 3, and 4), with a tandem repeat size of 12 base pairs. Lane6: 50 base pair ladder; Lane 1, 2, and 3: Amplicon obtained based on 4774 locus with fragment length of 250 bp (3tr) ; Lane 4, and 5: negative and positive control respectively. (e) MLVA typing of DNA from grown *S. enteritides* isolates using the 4301 locus (Lane 2, 3, and 4), with a tandem repeat size of 3 base pairs. Lane 1: 50 base pair ladder; Lane 2, 3 and 4: Amplicon obtained based on 4301 locus with fragment length of 175 bp (10tr), 175 (10tr), and 181 bp (12tr) respectively; Lane 5, and 6: negative and positive control respectively. (f) MLVA typing of DNA from grown *S. ententides* isolates using the 2053 locus (Lane 1, 2, and 3), with a tandem repeat size of 12 base pairs. Lane 6: 50 base pair ladder; Lane 1, 2 and 3: Amplicon obtained based on 2053 locus with fragment length of 196 bp (2tr), 184 bp (1tr), and 208 bp (3tr) respectively; Lane 4, and 5: negative and positive control respectively.

**Figure-3:**
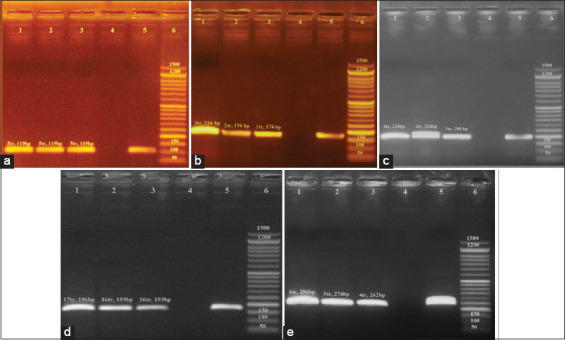
The results of multilocus variable number of tandem repeat analysis typing, based on DNA extracted from grown *Salmonella* Typhimurium isolates. (a) MLVA typing of DNA from grown *S. typhimurium* isolates using the 0681 locus (Lane 1, 2, and 3), with a tandem repeat size of 6 base pairs. Lane 6: 50 base pair ladder; Lane 1, 2 and 3: Amplicon obtained based on 0681 locus with fragment length of 119 bp (5tr) Lane 4, and 5: negative and positive control respectively. (b) MLVA typing of DNA from grown *S. typhimurium* isolates using the 0764 locus (Lane 1, 2, and 3), with a tandem repeat size of 20 base pair 6: 50 base pair ladder; Lane 1, 2 and 3: Amplicon obtained based on 0764 locus with fragment length of 214 bp (3tr), 194 bp (2tr), and 174 bp (1tr) respectively; Lane 4, and 5: negative and positive control respectively. (c) MLVA typing of DNA from grown *S . typhimurium* isolates using the 2053 locus (Lane 1, 2, and 3), with a tandem repeat size of 12 base pairs. Lane 6: 50 base pair ladder; Lane 1, 2 and 3: Amplicon obtained based on 2053 locus with fragment length of 220 bp (4tr), 220 bp (4tr), and 208 bp (3tr) respectively; Lane 4, and 5: negative and positive control respectively. (d) MLVA typing of DNA from grown *S. typhimurium* isolates using the 4301 locus (Lane 1, 2, and 3), with a tandem repeat size of 3 base pairs. Lane 6: 50 base pair ladder; Lane 1,2 and 3: Amplicon obtained based on 4301 locus with fragment length of 196 bp (17tr), 193 bp (16tr) and 193 bp (16tr) respectively; Lane 4, and 5: negative and positive control respectively. (e) MLVA typing of DNA from grown *S. typhimurium* isolates using the 4774 locus (Lane 1, 2, and 3), with a tandem repeat size of 12 base pairs. Lane 6: 50 base pair ladder; Lane 1, 2 and 3: Amplicon obtained based on 4774 locus with fragment length of 286 bp (6tr), 274 bp (5tr), and 262 bp (4tr) respectively; Lane 4, and 5: negative and positive control respectively.

Data for all six loci (0681, 0764, 0789, 2053, 4301, and 4774) were found to be polymorphic in both *S*. Enteritidis and *S*. Typhimurium DNA. A representative agarose gel is shown in Figures-2 and 3. All MLVA types were comprised single isolates with unique genotypes (Table-4) and yielded the results (Table-2), we expected based on a previous report [31]. The length of the yielded amplicon in bp must be calculated as number of repeats (Table-2).

## Discussion

In recent decades, the *S. enterica* serovars enteritidis and Typhimurium have been considered the main source of outbreaks in Egyptian poultry farms [33]. The use of effective and accurate molecular typing techniques has significantly benefited epidemiological studies. To prevent and detect potential outbreaks, further exploration of transmission methods is necessary. For this purpose, MLVA was recently introduced in Egyptian laboratories to investigate outbreaks of both *S*. Enteritidis and *S*. Typhimurium serovars. However, some economical and technical obstacles, including economic instability, higher cost of advanced molecular genotyping techniques, and the lack of adequate laboratory equipment for of characterization of *S*. Enteritidi*s* and *S*. Typhimurium, prevent widespread use of MLVA in Egypt.

This study documented the occurrence of *S*. Enteritidis and *S*. Typhimurium in Egyptian chickens. The findings are of public health concern, especially in the realm of poultry farming. The results revealed that 75% of the isolates submitted for PCR analysis were *S*. Enteritidis while the remaining 25% were *S*. Typhimurium. The serovars were distinguished through the amplification of two distinct primer sets. The enteritidis specific primer (*IE-1*) was reported previously [31,34]. The Typhimurium specific primer (*Flic-C*) amplified a gene that participates in flagellin synthesis and was chosen from a whole-genome sequence database for the *S*. Typhimurium strain AY649720 retrieved from GenBank [29,35]. The specificity of primer pairs was confirmed using the BLAST algorithm at NCBI (http://www.ncbi.nlm.nih.gov). The results of PCR amplification showed sharp bands of a 316-bp fragment (diagnostic for *S*. Enteritidis) and a 432-bp fragment (diagnostic for *S*. Typhimurium).

MLVA was assessed as a molecular tool for genotyping 12 samples of *Salmonella* DNA. Of those 12 samples, nine samples were *S*. Enteritidis and three were *S*. Typhimurium. DNA was extracted from isolates based on traditional epidemiological tracing information using six VNTR markers. These markers comprised Sal02, Sal04, Sal06, Sal10, Sal20, and Sal23 from a previously reported MLVA scheme developed for *S. enterica* [30].

There were 12 unique MLVA genotypes (A, B, C, D, E, F, G, H, and I) that represented *S*. Enteritidis. Alternatively, three unique genotypes (J, K, and L) represented *S*. Typhimurium. Tyhpimurium and Enteritidis genotypes were dispersed throughout the region of study area.

All six loci had polymorphisms in both *S*. Enteritidis and *S*. Typhimurium**.** The analysis of *S*. Enteritidis DNA revealed that five loci (0681, 0764, 0789, 2053, and 4774) were involved in only a 1-step change in TR copy number. The locus 4301 did exhibit a change in TR copy number, which varied from 8 to 14 copies with a 1- or 2-step change in the TR copy number. *S*. Typhimurium had a 1-step change only in the TR copy number in all loci examined.

This data could not be compared with other Egyptian studies as there have been no previous studies on the genotypes of *S*. Enteritidis and *S*. Typhimurium in Egypt. However, our results are similar to those reported from *S. enterica* isolated from blood and stool samples from symptomatic patients in France [30]. In the French study, loci Sal04, Sal06, Sal10, Sal20, and Sal23 showed higher diversity in tandem repeat copy number than in our study. Notably, there was a complete difference in the tandem repeat copy number of Sal 02. Another study based on the typing of Heidelberg isolates [36] revealed that tandem repeat copy numbers for Sal02, Sal10, and Sal20 are similar to our reported results for *S*. Enteritidis. This suggests the possibility that not all Sal loci are informative for the identification of *S. enterica* serovars. Therefore, the formation of a locus standardization model for each serovar is an urgent necessity. The data from molecular typing have allowed us to discern the genotypes of *S*. Enteritidis and *S*. Typhimurium that are prevalent in Egyptian chickens. Moreover, this epidemiological information gives us insight into how to prevent disease transmission of salmonellosis [37].

## Conclusion

MLVA typing is an effective and beneficial tool for investigating and identifying early warning signs of *Salmonella* outbreaks and providing epidemiological surveillance for *S*. Enteritidis and *S*. Typhimurium infections.

## Authors’ Contributions

SME created the research and experimental design, performed the laboratory experiment, data analysis, and wrote the manuscript. AAAE supervised the experiment, checked the data analysis, and revised the manuscript. WMMH supervised the experiment, helped in the laboratory work, and revised the manuscript. All authors contributed to drafting and revision of the manuscript. All authors read and approved the final manuscript.
